# MFS multidrug transporters in pathogenic fungi: do they have real clinical impact?

**DOI:** 10.3389/fphys.2014.00197

**Published:** 2014-05-28

**Authors:** Catarina Costa, Paulo J. Dias, Isabel Sá-Correia, Miguel C. Teixeira

**Affiliations:** Biological Sciences Research Group, Department of Bioengineering, Instituto Superior Técnico, IBB - Institute for Biotechnology and Bioengineering, Universidade de LisboaLisbon, Portugal

**Keywords:** multidrug resistance efflux pumps, drug:H+ antiporters, antifungal drug resistance, pathogenic fungi, *Candida* species

## Abstract

Infections caused by opportunistic fungal pathogens have reached concerning numbers due to the increase of the immunocrompromised human population and to the development of antifungal resistance. This resistance is often attributed to the action of multidrug efflux pumps, belonging to the ATP-binding cassette (ABC) superfamily and the major facilitator superfamily (MFS). Although many studies have focused on the role of ABC multidrug efflux transporters, little is still known on the part played by the Drug:H^+^ Antiporter (DHA) family of the MFS in this context. This review summarizes current knowledge on the role in antifungal drug resistance, mode of action and phylogenetic relations of DHA transporters, from the model yeast *S. cerevisiae* to pathogenic yeasts and filamentous fungi. Through the compilation of the predicted DHA transporters in the medically relevant *Candida albicans, C. glabrata, C. parapsilosis, C. lusitaniae, C. tropicalis, C. guilliermondii, Cryptococcus neoformans*, and *Aspergillus fumigatus* species, the fact that only 5% of the DHA transporters from these organisms have been characterized so far is evidenced. The role of these transporters in antifungal drug resistance and in pathogen-host interaction is described and their clinical relevance discussed. Given the knowledge gathered for these few DHA transporters, the need to carry out a systematic characterization of the DHA multidrug efflux pumps in fungal pathogens, with emphasis on their clinical relevance, is highlighted.

## Introduction

The multidrug resistance (MDR) phenomenon, characterized by the simultaneous acquisition of resistance to chemically and structurally different compounds (Sá-Correia et al., 2009; Morschhauser, 2010), poses a severe problem in the treatment of fungal infections. This is particularly relevant since the number of infections caused by opportunistic fungal pathogens has increased considerably in recent years due to the widespread use of antifungal drugs in immunocompromised patients, such as individuals undergoing chemotherapy, HIV-infected, or AIDS patients (Morschhauser, 2010).

There are mainly four mechanisms by which a cell can deal with a toxic compound: (i) drug target alteration, (ii) drug inactivation, (iii) reduced uptake, or (iv) active extrusion (Ernst et al., 2010). The latter occurs mainly due to the action of membrane transporters which belong to one of two superfamilies in fungi: the ATP-binding cassette superfamily (ABC) and the major facilitator superfamily (MFS) (Cannon et al., 2009; Sá-Correia et al., 2009; Morschhauser, 2010). The role of the ABC multidrug transporters in antifungal resistance in clinical isolates has been well characterized in the past decades. However, much less attention has been drawn to the expected role of the Drug:H^+^ Antiporter (DHA) family of the MFS.

In this paper, knowledge gathered so far on the role of the DHA family in antifungal drug resistance is reviewed, with emphasis on its clinical relevance. Although starting from what was found, in this context, in the model yeast *Saccharomyces cerevisiae*, particular focus is given to the DHA transporters found to occur, based on phylogenetic analysis, in pathogenic yeasts of the *Candida* genus and also in *Cryptococcus neoformans*, and *Aspergillus fumigatus*. Current challenges and expected impact of research in this topic is finally discussed.

## The Drug:H^+^ antiporter family: lessons from *Saccharomyces cerevisiae*

Upon the release of the complete *Saccharomyces cerevisiae* genome sequence (Goffeau et al., 1996), a total of 22 transporters belonging to the MFS were identified and clustered into two families: the drug:H^+^ antiporter family 1 (DHA1) and 2 (DHA2). These families differ mainly in the number of transmembrane spans (TMS), with the first having 12 and the second 14 TMS (Sá-Correia et al., 2009).

*S. cerevisiae* has 12 DHA1 and 10 DHA2 transporters, most of which have been implicated in MDR, while some are yet to be characterized (reviewed in Sá-Correia et al., 2009). Most of these transporters were found to confer resistance to a large number of unrelated chemicals. Given this apparent promiscuity, their exact mode of action as multidrug resistance determinants is controversial. For most *S. cerevisiae* DHA transporters a role in the physiology of the cell was further identified. That is the case of ScTpo1-4 and ScQdr3, which confer resistance to toxic levels of polyamines (Tomitori et al., 2001; Albertsen et al., 2003; Teixeira et al., 2011), of ScDtr1, that facilitates the translocation of bisformyl dityrosine through the prospore membrane during spore wall maturation (Felder et al., 2002), of ScQdr2, involved in potassium homeostasis (Vargas et al., 2007), and of ScAqr1, that has been proposed to excrete amino acids, such as homoserine and threonine (Velasco et al., 2004).

Most of the characterized transporters of *S. cerevisiae*, both from DHA1 and DHA2 subfamilies, confer resistance to more than one different growth inhibitory compound, with ScTpo1 being, by far, the one with the broadest range of predicted substrates (Sá-Correia et al., 2009). Among these compounds, some are of particular relevance in the combat against fungal phytopathogens. For example, ScFlr1 was found to confer resistance against the agricultural fungicides benomyl (Brôco et al., 1999) and mancozeb (Teixeira et al., 2008, 2010). The most interesting finding, however, is that, even though this is a non-pathogenic species, a lot of these efflux pumps confer resistance to or are up-regulated by widely used clinically relevant antifungal drugs, such as fluconazole (ScFlr1, ScQdr1, ScYhk8, and ScAzr1) (Tenreiro et al., 2000; Nunes et al., 2001; Barker et al., 2003), ketoconazole (ScAqr1, ScQdr1, ScQdr2, ScAzr1) (Tenreiro et al., 2000, 2002; Nunes et al., 2001), itraconazole (ScYhk8) (Barker et al., 2003) and caspofungin (ScTpo1) (Markovich et al., 2004). Some of them even confer resistance to more than one of these antifungal agents, as is the case of ScAzr1 and ScQdr1.

The paradigmatic case of the study of DHA transporters in *S. cerevisiae* highlights three important facts: (1) the existence and, thus, function of 20 out of 22 of these transporters remained concealed until the genome sequence was disclosed; (2) these transporters were indeed found to confer multidrug resistance, but also to play important roles in *S. cerevisiae* physiology; and (3) there is significant functional overlap between these transporters, making the discovery of their function a bigger challenge. The functional analysis of the *S. cerevisiae* DHA transporters provides clues on the function of homolog transporters from pathogenic yeast and filamentous fungi, but also suggests that their role tends to be elusive.

## Drug:H^+^ antiporter family: dissemination in pathogenic yeast and fungi

**Figure 1:**
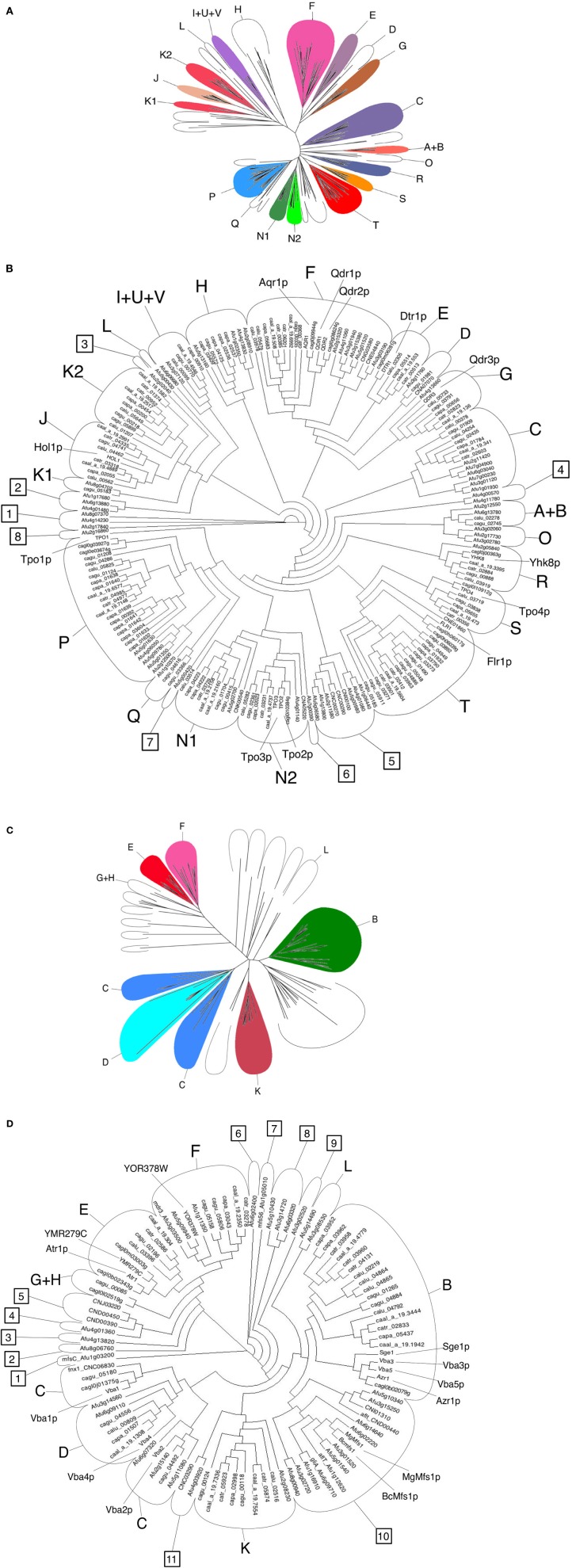
**Phylogenetic analysis of DHA1 and DHA2 transporters gathered from the *S. cerevisiae* S288C reference strain and from eight clinically important fungal species - *C. albicans, C. tropicalis, C. parapsilosis, C. guilliermondii, C. lusitaniae, C. glabrata, A. fumigatus* and *C. neoformans* - using the PROTDIST/NEIGHBOR packages of PHYLIP suite, as detailed in (Dias and Sá-Correia, 2013, in press). (A)** Radial phylogram showing the amino acid sequence similarity distances between these 197 full-size DHA1 transporters. **(B)** Circular cladogram showing the corresponding tree topology. The DHA1 proteins are distributed into 20 known phylogenetic clusters, labeled using letters and based on previous cluster annotation (Dias and Sá-Correia, in press), and 8 new phylogenetic clusters (clusters 1–4 and 6–7) comprising only members of the filamentous fungi. **(C)** Radial phylogram showing the amino acid sequence similarity distances between these 95 full-size DHA2 transporters. **(D)** Circular cladogram showing the corresponding tree topology. The DHA2 proteins are distributed into 8 known phylogenetic clusters, labeled using letters and based on a previous cluster annotation (Dias and Sá-Correia, 2013), and 11 new phylogenetic clusters (clusters 1–11) comprising only members of the filamentous fungi. The DHA1 and the DHA2 proteins encoded in the genome of *S. cerevisiae* S288c strain and biochemically characterized MgMfs1 and BcMfs1 DHA2 fungal proteins were used as functional reference and the corresponding names are indicated in the phylogenetic trees. The ARN and GEX proteins (Dias and Sá-Correia, 2013), were not included in this analysis.

The *Candida* DHA1 and DHA2 transporters considered in this review are those predicted to be encoded in the genomes of *C. albicans, C. tropicalis, C. parapsilosis, C. guilliermondii, C. lusitaniae*, and *C. glabrata* (Dias et al., 2010; Dias and Sá-Correia, 2013, in press). The DHA1 and DHA2 proteins encoded in the genomes of *A. fumigatus* and *C. neoformans* were identified using the annotation provided by the *Aspergillus* Genome Database (AspGD—http://www.aspgd.org/) and, based on the BlastP algorithm, using the *S. cerevisiae* DHA1 and DHA2 proteins to query the Genbank database, respectively. A total of 185 full-size DHA1 proteins and 85 full-size DHA2 proteins were found to be encoded in these eight fungal species, and clustered according to the phylogenetic trees displayed in Figure 1.

This review considers DHA1 proteins belonging to 20 previously reported phylogenetic clusters, labeled A to V (Dias et al., 2010; Dias and Sá-Correia, in press), and describes the occurrence of 8 new clusters, which include only *A. fumigatus* genes (clusters 1–4 and 6–7) or *A. fumigatus* and *C. neoformans* genes (cluster 5) (Figure 1). It becomes clear that the *A. fumigatus* genome encodes a higher number and higher diversity of DHA1 proteins compared with hemiascomycetous genomes. However, *A. fumigatus* lacks homologs to the *S. cerevisiae* Dtr1, Hol1, Tpo4 and Flr1 transporters (Figure 1). The genome of the basidiomycete yeast *C. neoformans* comprises a total of 9 DHA1 proteins, including homologs of *S. cerevisiae* Qdr1/Qdr2/Aqr1, Tpo2/Tpo3 and Flr1 proteins, one homolog of the *C. albicans* Tmp1/Tmp2 proteins and three proteins residing in the new phylogenetic cluster 5 (Figure 1).

The DHA2 proteins considered or identified in this study are distributed throughout 8 previously reported phylogenetic clusters (Dias and Sá-Correia, 2013), plus 11 newly defined clusters, composed only by *A. fumigatus* and/or *C. neoformans* proteins (labeled 1–11). The *A. fumigatus* genome encodes a total of 33 DHA2 transporters, two thirds of which reside in clusters lacking hemiascomycetous DHA2 members. *A. fumigatus* genome does not include homologs of Sge1/Azr1/Vba3/Vba5 or Atr1/YMR279C proteins. A total of 7 DHA2 proteins were found in the *C. neoformans* genome, among which only one *S. cerevisiae* homolog is found, clustering with Vba1/Vba2 (Figure 1).

Altogether, the abundance of predicted DHA transporters in the *Candida, Cryptococcus* and *Aspergillus* species considered, reaching 87 for *A. fumigatus*, is very high, strongly suggesting that these transporters must play an important function in these organisms. Among almost 300 ORFs compiled in Figure 1 only 12 have already been characterized, corresponding to around 5% of the total number. Their predicted involvement in drug resistance highlights the importance of characterizing all of them in a systematic way.

## Role of DHA transporters in antifungal drug resistance

Only a few DHA transporters have been linked to antifungal drug resistance in pathogenic fungi (Table 1). This appears to be mostly due to lack of characterization efforts. The *C. albicans* drug efflux pump Mdr1 was the first protein identified as a multidrug MFS transporter in a pathogenic fungus. CaMdr1 expression was found to confer resistance to fluconazole and ketoconazole, but not to itraconazole or miconazole, in *S. cerevisiae*, while overexpression in *C. albicans* leads to fluconazole resistance (Goldway et al., 1995; Hiller et al., 2006). CaNag3 and CaNag4 have also been shown to confer resistance to several antifungal drugs, such as cycloheximide, 4-nitroquinoline-*N*-oxide and 1–10 phenanthroline, being thus proposed to be multidrug efflux pumps (Yamada-Okabe and Yamada-Okabe, 2002). The *C. albicans* DHA1 transporter CaFlu1 was found to complement fluconazole hypersusceptibility in a *S. cerevisiae Δpdr5* mutant, but not to have a significant role in fluconazole resistance in *C. albicans* (Calabrese et al., 2000). On the other hand, Flu1 was more recently shown to confer resistance to the salivary human antimicrobial peptide histatin 5, playing a direct role in its efflux from *C. albicans* cells, thus reducing histatin 5 toxicity (Li et al., 2013). Of a total of 26 DHA transporters found to be encoded by *C. albicans* genome, 18 are still uncharacterized.

**Table 1:** **DHA1 and DHA2 transporters predicted from the genome sequences of *Candida* spp., *Aspergillus fumigatus*, and *Cryptococcus neoformans***.

**Species**	**Total #**	**Characterized #**	**Characterized ORFs**	**Description and role in Drug/Stress resistance or virulence**
	**Drug:H^+^ Antiporter 1 (DHA1) family members**			
*C. albicans*	18	7	*orf19.5604/MDR1*	Methotrexate is preferred substrate; overexpression in drug-resistant clinical isolates confers fluconazole resistance; repressed in young biofilms
			*orf19.6577/FLU1*	Involved in histatin 5 efflux
			*orf19.2158/NAG3*	Required for wild-type mouse virulence and cycloheximide resistance; Spider biofilm repressed
			*orf19.2160/NAG4*	Required for wild-type mouse virulence and wild-type cycloheximide resistance
			*orf19.508/QDR1*	Involved in biofilm architecture and thickness and virulence
			*orf19.6992/QDR2*	Involved in biofilm architecture and thickness and virulence
			*orf19.136/QDR3*	Involved in biofilm architecture and thickness and virulence
*C. glabrata*	10	4	*CAGL0J09944g/AQR1*	Involved in resistance to flucytosine, imidazoles, and acetic acid
			*CAGL0H06017g/FLR1*	Confers resistance to benomyl; gene is downregulated in azole-resistant strain
			*CAGL0I10384g/TPO3*	Confers imidazole and triazole drug resistance; involved in polyamine homeostasis; activated by CgPdr1
			*CAGL0G08624g/QDR2*	Confers imidazole drug resistance, involved in clotrimazole efflux; activated by CgPdr1p; upregulated in azole-resistant strain
*C. parapsilosis*	28	0		
*C. lusitaniae*	17	0		
*C. tropicalis*	18	0		
*C. guilliermondii*	31	0		
*C. neoformans*	9	1	*CNA07070*	Dityrosine transporter
*A. fumigatus*	54	0		
	**Drug:H^+^ Antiporter 2 (DHA2) family members**			
*C. albicans*	8	1	*orf19.2350*	Affects filamentous growth
*C. glabrata*	5	0		
*C. parapsilosis*	6	0		
*C. lusitaniae*	7	0		
*C. tropicalis*	8	0		
*C. guilliermondii*	11	0		
*C. neoformans*	7	2	*CNC03290*	Tetracycline efflux protein
			*CND00440/aflT*	Aflatoxin efflux pump
*A. fumigatus*	33	2	*Afu6g09710/gliA*	Predicted glioxin transporter
			*Afu1g05010/mfs56*	Mutation causes increased azole sensitivity

*The total predicted number and the number of characterized DHA transporters are accounted for. The description and role in drug/stress resistance or virulence of the few characterized DHA is highlighted (adapted from CGD and AspGD)*.

Close homologs to CaMdr1 were found in both *C. dubliniensis* and *C. tropicalis*. In the first, inactivation of the gene was shown to result in an increased susceptibility to fluconazole (Wirsching et al., 2001), while in the latter its expression increased when cells were treated with increasing concentrations of fluconazole and, at the same time, developed cross-resistance to azoles and terbinafine (Barchiesi et al., 2000).

*C. glabrata*, a pathogenic yeast that is phylogenetically closer to *S. cerevisiae* than to other *Candida* species, shows a high level of intrinsic resistance to fluconazole. However, the DHA transporter CgFlr1, a CaMdr1 homolog, was found to confer resistance to benomyl, in a strain that was already a mutant for both *CDR1* and *CDR2* genes, but not to fluconazole or other azoles (Chen et al., 2007). More recently, three additional DHA1 transporters were characterized in what concerns their contribution to the MDR phenomenon and their role in antifungal drug resistance. CgQdr2, CgAqr1, and CgTpo3 were found to be involved in multidrug resistance as they confer resistance to a wide variety of toxic compounds (Costa et al., 2013a,b, 2014), the most relevant being, for CgQdr2, the imidazoles clotrimazole, miconazole, tioconazole, and ketoconazole (Costa et al., 2013b), for CgAqr1, the antifungal drugs flucytosine and, less significantly, clotrimazole (Costa et al., 2013a) and for CgTpo3 the azole antifungals clotrimazole, ketoconazole, miconazole, tioconazole, itraconazole, and fluconazole (Costa et al., 2014). The role of CgAqr1 and CgTpo3 transporters in acetic acid (Costa et al., 2013a) and polyamine (Costa et al., 2014) resistance, respectively, is also an interesting feature given that *Candida* species have often to thrive in acetic acid and polyamine rich environments such as the vaginal mucosa or the urogenital tract, respectively.

For *Aspergillus fumigatus*, the transporter Mfs56 from the DHA2 family has been characterized and found to have a role in the resistance to itraconazole, posaconazole and ravuconazole (Bowyer et al., 2012). Also, the AfMdr3 transporter has been found to be overexpressed in cells treated with amphotericin B (Gautam et al., 2008). Interestingly, two characterized multidrug transporters from the phytopathogenic fungi *Botrytis cinerea*, Bcmfs1 (Hayashi et al., 2002), and *Mycosphaerella graminicola*, Mgmfs1 (Roohparvar et al., 2007), are shown to cluster together with cluster 10 (Figure 1). Mgmfs1 in particular was found to confer resistance to azoles and cycloheximide, among many other tested compounds (Roohparvar et al., 2007), suggesting that a similar function may be performed by the remaining members of the DHA2 cluster 10.

## Drug:H^+^ antiporter clinical impact: what's known and what is there to be found

The clinical impact of the DHA transporters in fungal pathogens is yet to be fully investigated. So far, only a couple of them have been shown to effectively contribute to the development, progression, or persistence of the infection in the host (Table 1). That is the case of *C. albicans* Mdr1, which has been found to be consistently expressed in high values in fluconazole resistant clinical isolates (Wirsching et al., 2000a,b), the same being observed for *C. dubliniensis* Mdr1 (Wirsching et al., 2001). CaNag3 and CaNag4 have also been shown to be involved in *C. albicans* virulence, as adult mice infected intravenously with 10^6^ or 10^7^ cells of the *Δcanag3, Δcanag4* or *Δcanag3canag4* mutants lived for at least 4 weeks (in the lowest cell concentration for single mutants, and both concentrations for double mutant), compared with a maximum of 11 days for the lowest concentration of the wild type (Yamada-Okabe and Yamada-Okabe, 2002). Very recently, the deletion of *C. albicans* Qdr1, Qdr2, and Qdr3 transporters was found to lead to defects in biofilm architecture and thickness and to attenuate virulence in a mouse model (Shah et al., 2014). Although the exact mechanism underlying this observation was not clarified, the expression of these transporters was found to have a deep effect in membrane lipid composition, which may underly the observed phenotypes (Shah et al., 2014). The *C. glabrata* DHA transporters CgAqr1 and CgTpo3 were also suggested to contribute to the survival of this pathogen within the host, as they confer resistance to acetic acid (Costa et al., 2013a) and polyamines (Costa et al., 2014), respectively, which accumulate to inhibitory concentrations in *Candida* infection prone environments. Indeed, the concentration of lactic or acetic acid can reach up to 125 mM in the vaginal tract, particularly under bacterial vaginosis (Chaudry et al., 2004), whereas polyamine concentrations can reach up to 15 mM spermine, 5 mM spermidine, and 3 mM putrescine in the urogenital tract (Tyms, 1989).

Of the high number of MFS transporters that fungal genomes encode, only a reduced amount has been shown to be involved in MDR or to be able to confer resistance to antifungal drugs, and an even lower number has been described as clinically relevant, so far. The lack of information on the clinical impact of fungal DHA transporters may be associated with the types of experiments carried out in this context. For instance, in *C. albicans*, the upregulation of the ABC transporters *CaCDR1* and *CaCDR2* in fluconazole-resistant isolates, which has used to assess their clinical relevance, was assessed mostly by targeted gene expression analysis (Sanglard et al., 1995, 1997), or in a few cases by the genome-wide analysis of the transcriptional response to antifungal drugs (Karababa et al., 2004; Liu et al., 2007). Furthermore, the impact of these transporters may not depend merely on their over-expression. For example, polymorphic mutant alleles of *CaMDR1* have been found among clinical isolates, inducing distinct drug resistance profiles (Gupta et al., 1998). More detailed analysis of the expression and sequence variation of DHA encoding genes in clinical isolates is likely to provide clues on their impact in the acquisition of MDR in clinical settings.

## Conclusions and perspectives

Given their predicted role in antifungal drug resistance, a few DHA transporters in pathogenic yeasts and fungi have already been characterized. So far, their characterization suggested interesting roles in antifungal drug resistance and, in some cases, in host-pathogen interactions. However, only 5% of the predicted DHA transporters in the 8 fungal pathogens considered in this review have ever been studied. It appears, thus, crucial to study this underappreciated family of transporters, as they may provide decisive insights into the mechanisms underlying antifungal drug resistance that may guide more efficient fungal infection diagnosis, prophylaxis, and therapeutics. Furthermore, unlike what has been observed for ABC drug efflux pumps, which are widespread from bacteria to man, the DHA family appears to be strictly conserved within bacteria and fungi, turning these proteins into interesting candidates as targets for the development of new antifungal drugs.

### Conflict of interest statement

The authors declare that the research was conducted in the absence of any commercial or financial relationships that could be construed as a potential conflict of interest.
